# The influence of personality on the effect of iTBS after being stressed on cortisol secretion

**DOI:** 10.1371/journal.pone.0223927

**Published:** 2019-10-16

**Authors:** Matias M. Pulopulos, Sara De Witte, Marie-Anne Vanderhasselt, Rudi De Raedt, Johan Schiettecatte, Ellen Anckaert, Alicia Salvador, Chris Baeken

**Affiliations:** 1 Department of Experimental Clinical and Health Psychology, Ghent University, Ghent, Belgium; 2 Department of Head and Skin, Ghent University, Ghent, Belgium; 3 Ghent Experimental Psychiatry (GHEP) Lab, Ghent, Belgium; 4 Laboratory of Hormonology and Tumor Markers, University Hospital (UZBrussel), Brussels, Belgium; 5 Laboratory of Social Cognitive Neuroscience, Department of Psychobiology and IDOCAL, University of Valencia, Valencia, Spain; 6 Department of Psychiatry, University Hospital (UZBrussel), Brussels, Belgium; 7 Eindhoven University of Technology, Department of Electrical Engineering, Eindhoven, the Netherlands; Washington University, St. Louis, UNITED STATES

## Abstract

Over the last years, individualization of repetitive Transcranial Magnetic Stimulation (rTMS) parameters has been a focus of attention in the field of non-invasive stimulation. It has been proposed that in stress-related disorders personality characteristics may influence the clinical outcome of rTMS. However, the underlying physiological mechanisms as to how personality may affect the rTMS response to stress remains to be clarified. In this sham-controlled crossover study, after being stressed by the Trier Social Stress Test, 38 healthy females received two sessions of intermittent theta burst stimulation (iTBS) applied to the left dorsolateral prefrontal cortex. To take possible personality influences into account, they also completed the Temperament and Character Inventory. Mood and salivary cortisol were assessed throughout the experimental protocol. Overall, two iTBS sessions did not significantly alter mood or influenced cortisol secretion. When taking into account personality features, higher scores on the character dimension Cooperativeness was related to decreased cortisol output, only when active iTBS was administered after the social stressor. In line with other studies, personality features such as the character dimension Cooperativeness may be of particular interest to explain individual neurobiological responses to neurostimulation.

## Introduction

Repetitive transcranial magnetic stimulation (rTMS) is an approved clinical treatment for major depression [1]. Despite current efforts to increase clinical improvement, the underlying mechanisms of how and in whom rTMS can elevate depressed mood in a relatively short time span are still poorly understood. However, this could be crucial in the development of better treatment parameters [2,3]. Given that the clinical outcome of non-invasive stimulation paradigms still remains rather modest [4,5], a more personalized approach is of growing interest in the field of neurostimulation [6]. Amongst other approaches to individualize treatment parameters such as biotyping (e.g. [7]), personality features may be of interest to predict the efficacy to rTMS, in particular in stress-related disorders. Within this context, recent observations indicate that the Temperament and Character Inventory (TCI; [8,9] may be important to understand the differences in the rTMS response. According to the authors of the TCI, *character* refers to self-concepts and individual differences in goals and values, which are moderately influenced by socio-cultural learning. *Temperament* dimensions are thought to relate to the automatic emotional responses to experiences and are moderately heritable, remaining relatively stable throughout life [9]. In a refractory melancholic MDD sample we have recently demonstrated that higher scores on Self-Directedness—one of the three character dimensions of the TCI [8,9]—was related to the high frequency (HF)-rTMS treatment response [10]. Although methodological differences such as patient selection and stimulation parameters make studies difficult to compare, in two studies it has been demonstrated that some dimensions of the NEO-V model of personality [11] may also predict the outcomes of rTMS on MDD patients: Berlim et al. [12] reported that Extraversion predicted the clinical outcome of left (dorsolateral) prefrontal cortex (DLPFC) HF-rTMS treatment and McGirr et al. [13] found that Agreeableness and Conscientiousness were predictive for remission for deep HF-rTMS. Interestingly, De Fruyt et al. [14] demonstrated that Conscientiousness and Extraversion (NEO-V) are positively associated with Self-Directedness (TCI), and that Agreeableness and Extraversion (NEO-V) are positively correlated to Cooperativeness (TCI), also one of the three character dimensions of the TCI (the third character dimension of the TCI is Self-transcendence). Regarding temperament dimensions, Siddiqi et al. [15] have recently shown that higher Persistence scores predicted antidepressant response to rTMS in a broad range of patients with major depression. With respect to other TCI scales, Singh et al. [16] observed in healthy participants after a single HF-rTMS session that Harm Avoidance was related to a decrease in connectivity between the default network model and the subgenual Anterior Cingulate Cortex, a brain area that is assumed to be behind HF-rTMS treatment effects [17]. However, no previous studies have shown a relationship between this Harm Avoidance and clinical outcome after rTMS treatment [10,15].

These findings suggest that personality dimensions, and specially Cooperativeness, Self-Directedness, and Persistence may be used to predict the response to rTMS at the individual level. However, the use of subjective questionnaires to assess traits provides little insight into the underlying neurobiological mechanisms involved. Endocrinological responses (e.g., cortisol) operate rather independently of consciously experienced mood and could provide insight into the neurobiology of emotion processing in healthy as well in mentally affected states [18]. Importantly, Hori and colleagues [19] suggested the possibility of differentiating personality-related subtypes of depression based on different patterns of hypothalamic-pituitary-adrenal (HPA) axis regulation. Moreover, an important factor in the response of rTMS could be related to the endocrinological response of the HPA system [20]. Indeed, Keck [21] proposed that the influence of rTMS may occur at the hypothalamic level, suggesting that the DLPFC, the most common stimulated area in major depression, participates in the rTMS-induced blunted response of HPA-activity by inhibiting cortisol releasing hormone synthesis and release. In agreement with these assumptions, in a sample of severely depressed patients, salivary cortisol concentrations decreased immediately after one active left DLPFC HF-rTMS session and not after sham rTMS [22]. Pridmore et al. [23] also observed normalization of the dexamethasone suppression test in depressed subjects after multiple sessions of HF-rTMS. These observations suggest that the clinical effects of rTMS could be associated with a normalization of HPA-axis functioning, acting in a similar way as e.g. pharmacological interventions [24,25].

Also in non-depressed samples, the application of rTMS (single sessions) on the DLPFC has been shown to affect the HPA-system, however, only when the participants were being stressed [26,27], or when taking the individual characteristics related to stress (e.g., state anxiety) into account [28]. Therefore, to examine the effects of rTMS on the HPA-system in a healthy state, participants may need to be stressed in order to get as close as possible to the depressed state [27,29]. Furthermore, individual differences in stress sensitivity may influence cortisol secretion during non-invasive neurostimulation. Indeed, individual characteristics such as age, gender, or personality features are related to different aspects of HPA axis activity [30–35]. Along this line, Cooperativeness and Self-directedness have been associated with gray and white matter volume in the medial frontal cortices (Cooperativeness and Self-Directedness) and the anterior cingulate cortex (Self-directedness) [36], and Persistence has been related to activity of the lateral orbital and medial prefrontal cortex [37,38], areas that are closely connected to the DLPFC and that participate in the HPA-axis response to stress [39].

In this sham-controlled proof-of-concept study, we examined whether personality features would influence the effect of excitatory rTMS on HPA-system regulation in healthy volunteers in a stressed state. Previous research has shown that cortico-thalamic-limbic pathways related to depression and characterized by deactivation of dorsal areas, and increased activity of the amygdala can be observed in ‘a stressed’ brain (e.g., [40]). Thus, in this study, stressed healthy individuals are used as a model to investigate the influence of TCI on the effect of rTMS on the HPA axis. This investigation may provide important information to understand previous results in depressed patients and to guide future investigations. Because successive sessions of rTMS—more than one session daily—may have similar to better clinical outcomes in stress-related disorders [41,42], here we applied two successive rTMS sessions. Given that compared to excitatory HF-rTMS, excitatory intermittent theta burst stimulation (iTBS) matches clinical effects [43] or may even exceed brain activity processing [44], here we applied two iTBS sessions. In order to confidently induce acute stress, our participants performed the Trier Social Stress Test (TSST, [45]—which is the gold standard for examining the cognitive neurobiology of acute stress in humans [46]—before the two iTBS sessions. As gender and age could be a possible confounder in HPA-system regulation protocols [47], also with the TSST [33,48], and across psychiatric disorders [49], we chose to use a more ‘uniform’ group of female subjects in their young adulthood. Moreover, given that both the overall cortisol secretion during stressful situations and the stress-induced changes in cortisol levels are markers of HPA axis regulation [50,51], we investigated the effect of iTBS on the area under the curve with respect to the ground (*AUCg*, indexing overall cortisol secretion and reflecting the intensity of the cortisol response), and the area under the curve with respect to the increase (*AUCi*, indexing the stress-induced changes in cortisol levels and reflecting the sensitivity of the HPA axis to stressful events). Finally, to evaluate the effects of personality features on HPA-system regulation to the iTBS, in line with our former research on the influences of personality features on emotional processing in females [10,22,26,36,52–54], all participants were assessed with the TCI.

We hypothesized that after being stressed, we would find significant decreases in cortisol secretion after active iTBS as compared to sham. Considering previous results in patients with major depression showing that the HF-rTMS treatment response is associated with higher Self-Directedness [10], Persistence [15] and with personality dimensions that are highly correlated with both Self-Directedness and Cooperativeness [12, 13], we expected that especially these dimensions would influence the attenuation of cortisol secretion. Thus, we expected that individuals reporting higher scores on Self-Directedness, Cooperativeness, and Persistence will show lower HPA response after active iTBS, but not after sham stimulation. Previous studies did not observe an influence of the other TCI subscales on the effect of TMS on treatment response or HPA-axis activity. Therefore, we did not expect that these dimensions would have an influence on the effect of iTBS on cortisol secretion.

## Experimental procedures

### Participants

Forty healthy females in their young adulthood were recruited through student fora of Ghent University as well as social media complying to the following inclusion criteria: (a) no current/history of psychiatric disorders according the Mini-international Neuropsychiatric Interview (MINI; [55] based on DSM-IV and ICD-10 criteria, (b) Beck Depression Inventory (BDI-II-NL; [56], Dutch translation by Van der Does [57]) score below 14, (c) no current/history of neurological or cardiological problems, (d) no metal implanted objects in the body, (e) no current psychotropic medications, (f) right-handed and (g) female (not pregnant).

Two participants were excluded from the original sample after the screening on the first day, one because of a current psychiatric disorder and another because of neurological problems. All females used hormonal contraceptives. The final sample included in the analyses was composed of 35 participants (*M* age = 23.60 years, *SD* = 2.87, age range = 18–28 years) (see the *Results* section for a description of the exclusion of participants from the analyses). The study was approved by the ethics committee of the Ghent University hospital (UZGent) and is part of a larger project examining the effects of iTBS on stress. The influence of stress-related individual differences and functional connectivity on the effect of iTBS on stress induction will be published elsewhere. All gave written informed consent and were financially compensated for their participation.

### Assessment

#### Temperament and Character Inventory

Before the start of the study, all participants were assessed with the TCI [9], using a Dutch version of the TCI [58]. The TCI is 240-item questionnaire consisting of 4 temperament scales: Novelty Seeking (minimum score = 0, maximum score is 40), Harm Avoidance (minimum score = 0, maximum score is 36), Reward Dependence (minimum score = 0, maximum score is 24), Persistence (minimum score = 0, maximum score is 8), and three character scales: Self-Directedness (minimum score = 0, maximum score is 44), Cooperativeness (minimum score = 0, maximum score is 42) and Self-Transcendence (minimum score = 0, maximum score is 33)[9].

#### Visual analogue scales

Six horizontal 100 mm visual analogue scales (VAS; [59]) were used to detect subtle changes in mood. Feelings of ‘tiredness’, ‘vigor’, ‘anger’ ‘tension’, ‘depression’ and ‘cheerfulness’ were rated “*at this moment*”. The minimum score on each VAS subscale is 0, and the maximum score is 100. Subjects were asked to rate their mood at the end of the habituation phase (T_1_), immediately after the TSST (T_2_), after the first iTBS session (T_3_), before (T_4_) and after (T_5_) the second iTBS session, and five minutes after the second iTBS sessions (T_6_).

#### Cortisol

As in Baeken et al. [22,26–28], saliva samples were collected using a salivette (Sarstedt, Germany), with an insert containing a sterile polyester swab for collecting saliva, yielding a clear and particle-free sample. The salivettes were used according to the instructions provided by the manufacturer. Saliva cortisol levels (μg/L) were determined by Cortisol Saliva Luminescence immunoassay (IBL International GmbH, Germany). Limit of Quantification was 0.12 μg/L and the within-run and between-run variation coefficients were less than 5%. Furthermore, the intra-individual stability of baseline salivary cortisol levels is reported to be more stable in women [60]. To limit the influence of the circadian rhythm [61], all the sessions started in the afternoon. Salivary cortisol levels were measured at the end of the habituation phase (T_1_), after the preparation phase of the TSST (T_2_), immediately after the TSST (T_3_), after the first iTBS session (T_4_), before and after the second iTBS session (T_5_ and T_6_), and five minutes after the second iTBS session (T_7_).

#### Stress induction: Trier Social Stress Test (TSST)

To induce acute stress, a variant of the TSST [45] was used. In this variant, participants were positioned in front of a one-way mirror, so they could only see themselves. They were notified of a jury being present at the other side of the mirror, as they watched people going into this room before the TSST experiment. Using a connected sound system between the two rooms, the jury was able to talk to the participants. A camera was positioned in the room and participants were told their performances would be recorded for non-verbal communication and voice frequency analyses. Similar to the classical TSST, participants were first asked to perform a 5 min public speech, and they were informed that they had 3 minutes to prepare the speech. After the preparation phase, the participants performed the 5 min speech, and hereafter they were asked to perform a 5 min mental arithmetic discounting task.

#### Neurostimulation: iTBS

iTBS stimulation was applied using a Magstim Rapid2 Plus1 magnetic stimulator (Magstim Company Limited, Wales, UK) connected to a 70 mm figure-of-eight shaped coil. For the sham procedure, we used the Magstim 70mm Double Air Film sham coil. This coil is identical in all aspects to its active variant, but without stimulation output. By stimulating the peripheral nerves of the face and scalp, the Air Film sham coil looks, sounds and feels like an active coil, but it does not deliver active stimulation of cortical neurons. The sham coil was placed exactly on the same DLPFC location. Before the active and sham stimulation, the individual resting motor threshold (110%) was determined by inducing a motor evoked potential on the right abductor pollicis brevis muscle. For one iTBS session, the following parameters were used: frequency 50Hz, burst frequency 5Hz, 1620 pulses in total spread over 54 cycles in which each cycle includes 10 burst each 3 pulses with a train duration of 2 seconds and an inter-train interval of 6 seconds. These excitatory parameters are an exact copy as we used to treat depressed patients [41]. Following previous research investigating the effect of rTMS over the DLPFC on cortisol secretion [e.g., 22,26,27], we stimulated the mid-center of the left prefrontal gyrus (Brodmann 9/46). Using individual neuroanatomical MRI data, the left DLPFC was visually identified based on the subject own gyral morphology. The Brainsight neuronavigation system (BrainsightTM, Rogue Research, Inc) was used during the experiment to accurately place the active and sham coil in a perpendicular position to the DLPFC.

### Experimental protocol

**Fig 1:**
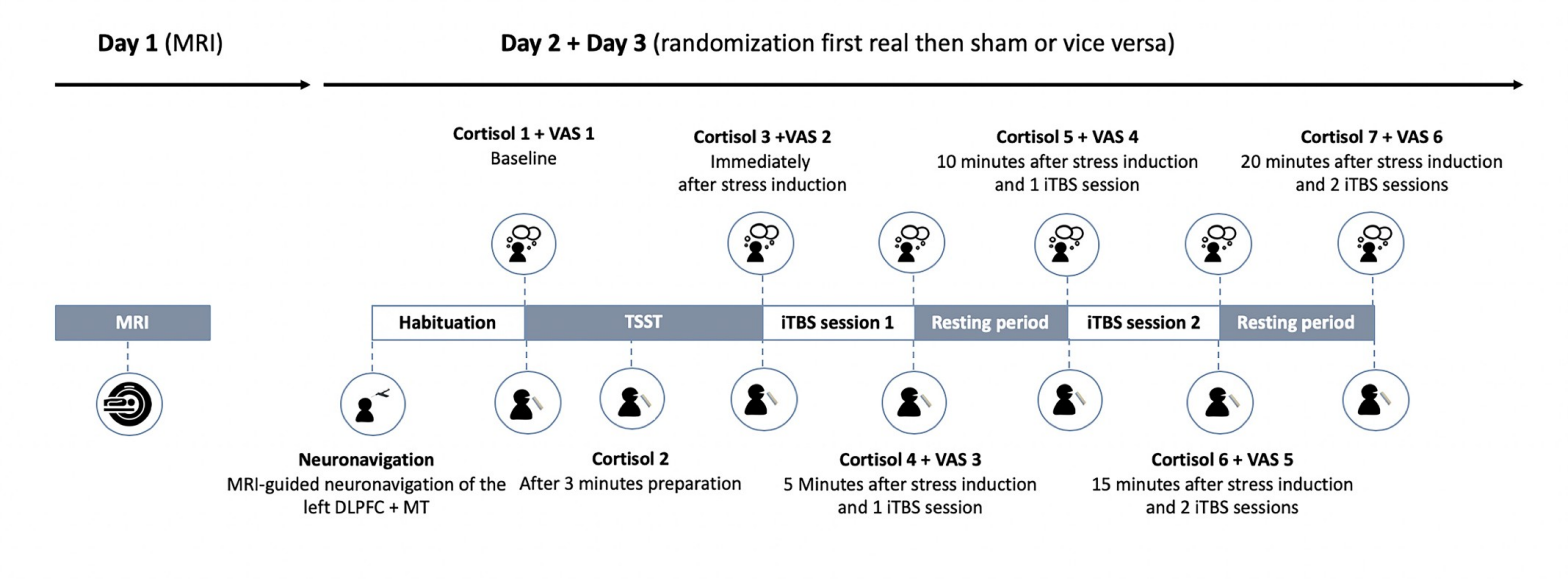
Overview of the protocol. MRI = Magnetic Resonance Imaging; MT = Motor Threshold; VAS = Visual Analogue Scale; TSST = Trier Social Stress Test; iTBS = intermittent Theta Burst Stimulation.

After inclusion, all participants were invited to the Ghent University Hospital on three separate days. First, to accurately locate the left DLPFC, we gathered the individual neuroanatomical data using a T1-weighted MRI scan in a Siemens 3T TrioTim MRI scanner (Siemens, Erlangen, Germany). Hereafter, participants were randomly assigned (computer) to a real-first or sham-first stimulation session. On each of both stimulation days, after 10 min of habituation, the participants performed the TSST. After the stress task, two iTBS sessions (both either active or sham) were applied to the left DLPFC with a five minute resting period in between (See Fig 1). Between stimulation days, to avoid carry-over effects, a time delay of at least one week was respected.

### Statistical analysis

All collected data were analyzed with SPSS 24 (Statistical Package for the Social Sciences). Where necessary, we applied the Greenhouse-Geisser correction to ensure the assumption of sphericity. The significance level was set at *p*<0.05, two-tailed, for all analyses.

To examine whether possible mood changes by the iTBS applications could influence our results, mood changes were analyzed with a mixed 2X6 repeated measures MANOVA. Within-subject factors were Stimulation (active vs. sham stimulation) and Time (T_1_, T_2_, T_3_, T_4_, T_5_, and T_6_). Order (1^st^ active-iTBS vs 1^st^ sham-iTBS) was the between-subjects factor. The six VAS mood scales (‘fatigued’, ‘vigorous’, ‘angry’, ‘tensed’, ‘depressed’ and ‘cheerful’) were the multiple dependent variables. Positive mood scales were reversed, indicating that higher scores referred to more negative affect.

For cortisol data, we computed the *AUCg* and *AUCi*, using the formulas proposed by Pruessner et al. [50]. The *AUCg* is an index of the total cortisol release by the HPA-axis, and it reflects the intensity of the stress response. The *AUCi* reflects the hormonal change over time and is considered an index of the sensitivity of the HPA-axis to the stressful event [51]. Importantly, paired *t-*tests showed that during both the sham and active-iTBS sessions cortisol levels increased from baseline to after the stress task (i.e., maximum cortisol levels after stress)(*p*<0.01), and that cortisol levels at baseline and the stress-induced increase in cortisol levels were similar in both sessions (*p*>0.72).

To investigate the effects of iTBS on *AUCg* and *AUCi*, we performed two mixed ANCOVAs using the active and sham *AUCg* and *AUCi* values as dependent variables, Stimulation (active-iTBS vs. sham-iTBS) as a within-subjects factor, and Order (1^st^ active-iTBS vs 1^st^ sham-iTBS) as a between-subjects factor. Given that cortisol secretion follows a circadian rhythm and the activity of the HPA-axis under stressful situations is affected by the time of the day [61], we controlled for the time when the participant started the two experimental sessions. Participants started the two sessions at a similar time (paired *t*-test: *t*(34) = 0.98, *p* = 0.332), and the mean of the starting time of the two stimulation sessions was used as a covariate in the ANCOVAs. In a second step, to investigate the influence of the TCI dimensions (i.e., Temperaments: Novelty Seeking, Harm Avoidance, Reward Dependence, Persistence; Characters: Self-Directedness, Cooperativeness, Self-Transcendence) to this experimental procedure, we performed a series of mixed ANCOVAs using the active and sham *AUCg* and *AUCi* values as dependent variables, Stimulation (active-iTBS vs. sham-iTBS) as the within-subject factor, Order (1^st^ active-iTBS vs 1^st^ sham-iTBS) as a the between-subjects factor, and we included the time when the participant started the sessions and each temperament or character dimension as covariate. Analyses were performed for each temperament and character scale separately. Importantly, the residuals of all the analyses showed a normal distribution (Kolmogorov-Smirnov: all *p*>0.13; Shapiro-Wilk: all *p*>0.11), and therefore, the analyses meet the assumption of normality. When analyses showed a statistically significant interaction between stimulation (active-iTBS vs. sham-iTBS) and a TCI dimension, partial correlations (including the time when the participants started the session as a covariate) were used to investigate the relationship between *AUCg* and *AUCi* for the sham and active iTBS sessions and the TCI dimension.

## Results

Nineteen participants first received active iTBS before sham, and the 19 other volunteers received sham iTBS followed by the active condition. Three participants were excluded from the analyses because 1) they did not complete all the questions of the TCI questionnaire (one participant), or 2) because the *AUCg* and *AUCi* could not be calculated due to missing data (two participants). One participant was considered as an outlier for *AUCi* data (-3SD) and was consequently excluded from the analyses with this variable. TCI scales and salivary cortisol data are summarized in Table 1.

**Table 1:** Cortisol values during the active and sham iTBS sessions, and TCI scales values.

Cortisol (μg/L)	Mean (SD)	TCI	Mean (SD)
**T1**	**Active**	1.02 (0.55)	***Temperaments***	
	**Sham**	1.05 (0.65)	**Novelty Seeking**	21.03 (6.13)
**T2**	**Active**	0.95 (0.52)	**Harm Avoidance**	12.63 (6.79)
	**Sham**	0.96 (0.51)	**Reward Dependence**	18.14 (3.13)
**T3**	**Active**	1.10 (0.70)	**Persistence**	4.63 (1.93)
	**Sham**	1.12 (0.70)	***Characters***	
**T4**	**Active**	1.28 (0.86)	**Self-Directedness**	34.60 (5.00)
	**Sham**	1.29 (0.85)	**Cooperativeness**	36.89 (4.14)
**T5**	**Active**	1.23 (0.96)	**Self-Transcendence**	7.80 (4.81)
	**Sham**	1.15 (0.77)		
**T6**	**Active**	1.13 (0.78)		
	**Sham**	1.02 (0.65)		
**T7**	**Active**	1.07 (0.71)		
	**Sham**	1.07 (0.65)		
***AUCg***	**Active**	4554.99 (459.41)		
	**Sham**	3864.30 (344.71)		
***AUCi***	**Active**	357.35 (352.54)		
	**Sham**	192.92 (242.43)		

Note: AUCg = Area under the curve with respect to the ground, *AUCi* = Area under the curve with respect to the increase, TCI = Temperament and Character Inventory.

### Mood effects

The repeated measures MANOVA revealed a significant main effect of Time (*F*(30,845) = 3.79, *p*<0.01, partial eta squared = 0.12) and a significant interaction between Stimulation and Order (*F*(6,29) = 4.48, *p* = 0.01, partial eta squared = 0.48). There were no other significant main or interaction effects. To follow up on the main effect of Time and the interaction between Stimulation and Order, we performed separate univariate ANOVAs. We observed a significant main effect of Time on the subscales ‘vigorous’ (*F*(3.61,116.67) = 3.09, *p*<0.05, partial eta squared = 0.08), ‘angry’ (*F*(2.90,98.53) = 2.96, *p*<0.05, partial eta squared = 0.08), ‘tensed’ (*F*(3.51,119.28) = 10.47, *p*<0.01, partial eta squared = 0.24), and ‘cheerful’ (*F*(3.22,109.41) = 4.13, *p*<0.01, partial eta squared = 0.11). Overall, participants got less cheerful during the protocol compared to when they arrived. Participants were more tensed after the TSST (at T_2_) compared to all other time points (all *p*<0.05). And participants were angrier immediately after the TSST (at T_2_) compared to after two sessions of stimulation (T_5_) (all *p*<0.05). Also, there was a significant interaction effect of Stimulation and Order on the subscales ‘angry’ (*F*(1,34) = 7.74, *p*<0.01, partial eta squared = 0.19) and ‘tensed’ (*F*(1,34) = 6.86, *p*<0.05, partial eta squared = 0.17). Showing that participants were overall (i.e., before and after the stimulation) angrier and more tense when they got active stimulation during the first session, whereas this was not the case when the active stimulation was during the second session. VAS mood ratings are summarized in Table 2.

**Table 2:** Mean and standard deviations for the Visual Analogue Scale (VAS) though the protocol (also see Fig 1). Scores are expressed on scales from 0 cm to 10 cm with a range of absence of the emotion to the max of the emotion.

		Fatigued	Vigorous	Angry	Tensed	Depressed	Cheerful
**T1**	**Active**	3.59 (2.13)	4.20 (2.12)	0.56 (0.89)	1.53 (1.62)	0.38 (0.59)	3.52 (1.92)
	**Sham**	3.41 (2.08)	4.20 (2.39)	0.46 (0.71)	1.50 (1.24)	0.26 (0.31)	3.27 (1.92)
**T2**	**Active**	3.62 (2.16)	4.37 (2.25)	0.80 (1.11)	2.44 (2.05)	0.29 (0.44)	4.02 (2.10)
	**Sham**	3.48 (2.43)	4.08 (2.12)	0.80 (1.20)	2.30 (2.12)	0.23 (0.25)	3.79 (2.19)
**T3**	**Active**	3.87 (2.03)	4.58 (2.34)	0.62 (1.16)	1.87 (1.89)	0.29 (0.33)	4.16 (2.22)
	**Sham**	3.70 (2.12)	4.55 (2.32)	0.36 (0.40)	1.60 (1.91)	0.24 (0.26)	3.73 (2.09)
**T4**	**Active**	3.79 (2.08)	4.43 (2.14)	0.66 (1.56)	1.44 (1.49)	0.20 (0.20)	4.08 (2.12)
	**Sham**	3.30 (2.22)	4.35 (2.13)	0.57 (0.94)	1.38 (1.91)	0.45 (1.26)	3.93 (2.30)
**T5**	**Active**	3.74 (2.21)	4.57 (2.40)	0.32 (0.46)	1.55 (1.91)	0.25 (0.25)	3.94 (2.22)
	**Sham**	4.16 (2.57)	4.59 (2.56)	0.41 (0.59)	1.32 (1.98)	0.37 (1.24)	3.86 (2.44)
**T6**	**Active**	3.83 (2.13)	4.11 (2.23)	0.32 (0.39)	1.05 (1.38)	0.22 (0.28)	4.06 (2.50)
	**Sham**	3.82 (2.61)	4.12 (2.49)	0.54 (1.11)	0.94 (1.77)	0.38 (1.22)	3.92 (2.50)

### Salivary cortisol and TCI

Regarding the effect of iTBS on cortisol, the results of the mixed ANCOVA for *AUCg* and separately for the *AUCi* values as dependent variables, Stimulation (active-iTBS vs. sham-iTBS) as a within-subjects factor, stimulation Order (1^st^ active-iTBS vs 1^st^ sham-iTBS) as a between-subjects factor, and the time when the session started as covariate showed no significant main effect of Stimulation for *AUCg* (*F*(1,32) = 0.11, *p* = 0.74, partial eta squared = 0.08) and *AUCi* (*F*(1,31) = 0.04, *p* = 0.84, partial eta squared<0.01), and a no significant main effect of order for *AUCg* (*F*(1,32) = 1.20, *p* = 0.28, partial eta squared = 0.04) and *AUCi* (*F*(1,31) = 0.01, *p* = 0.91, partial eta squared<0.01). The interaction between Stimulation and Order was not significant for *AUCg* (*F*(1,32) = 0.23, *p* = 0.63, partial eta squared = 0.01), but it was statistically significant for *AUCi* (*F*(1,31) = 8.48, *p*<0.01, partial eta squared = 0.22). Post hoc analyses revealed that during the first and second experimental sessions, there were no significant differences in *AUCi* scores between active-iTBS and sham-iTBS (1^st^ experimental session, active-iTBS vs sham-iTBS: *p* = 0.14; 2^nd^ experimental session, active-iTBS vs sham-iTBS: *p* = 0.14). Participants in the 1^st^ active-iTBS group showed a significant higher *AUCi* during the first session (i.e., active-iTBS) than during the second session (i.e., sham-iTBS) (*p* = 0.03). Participants in the 1^st^ sham-iTBS group showed a marginally significant higher *AUCi* during the first session (i.e., sham-iTBS) than during the second session (active-iTBS) (*p* = 0.08). The time when the participants started the session was a significant factor for *AUCg* (*F*(1,32) = 11.02, *p*<0.01, partial eta squared = 0.26), but not for *AUCi* (*F*(1,31) = 2.20, *p* = 0.15, partial eta squared = 0.07).

To investigate the effect of TCI dimensions, we performed *AUCg* and *AUCi* mixed ANOVAs with stimulation (active-iTBS vs. sham-iTBS) as the within-subjects factor, and Order (1^st^ active-iTBS vs 1^st^ sham-iTBS) as the between-subjects factor, and the TCI dimensions and the time when the session started as covariates. For all analyses, the main factors Stimulation and Order were not statistically significant (*F*(1,31)<3.13, *p*>0.09, partial eta squared<0.09). For all the analyses with *AUCg*, but not for the analysis with *AUCi*, the time when the participant started the session was a significant factor (*AUCg*: *F*(1,31)>8.11, *p*<0.01, partial eta squared>0.21; *AUCi*: *F*(1,30)<3.02, *p*>0.09, partial eta squared<0.09).

For the temperaments dimensions of the TCI, none of the analyses with *AUCg* and *AUCi* showed a significant main or interaction effect (*AUCg*: *F*(1,312)<1.38, all *p*>0.25, partial eta squared<0.04; *AUCi*: *F*(1,30)<1.26, all *p*>0.27, partial eta squared<0.04). For the analyses with the TCI characters dimensions and *AUCi*, none of the analyses showed a significant main or interaction effect (*AUCi*: *F*(1,30)<1.24, all *p*>0.28, partial eta squared<0.04). For the analyses with characters dimensions of the TCI and *AUCg*, the main factor Stimulation showed a significant interaction with Cooperativeness (*F*(1,31) = 4.67, *p*<0.05, partial eta squared = 0.12), and Self-Transcendence (*F*(1,31) = 6.87, *p* = 0.01, partial eta squared = 0.18), but not with Self-directedness (*F*(1,31) = 0.64, *p* = 0.43, partial eta squared = 0.02). We observed no significant main effects of Cooperativeness (*F*(1,31) = 3.48, *p* = 0.07, partial eta squared = 0.10), Self-Directedness (F(1,31) = 0.65, *p* = 0.43, partial eta squared = 0.02), and Self-transcendence (*F*(1,31) = 0.61, *p* = 0.44, partial eta squared = 0.02).

**Fig 2:**
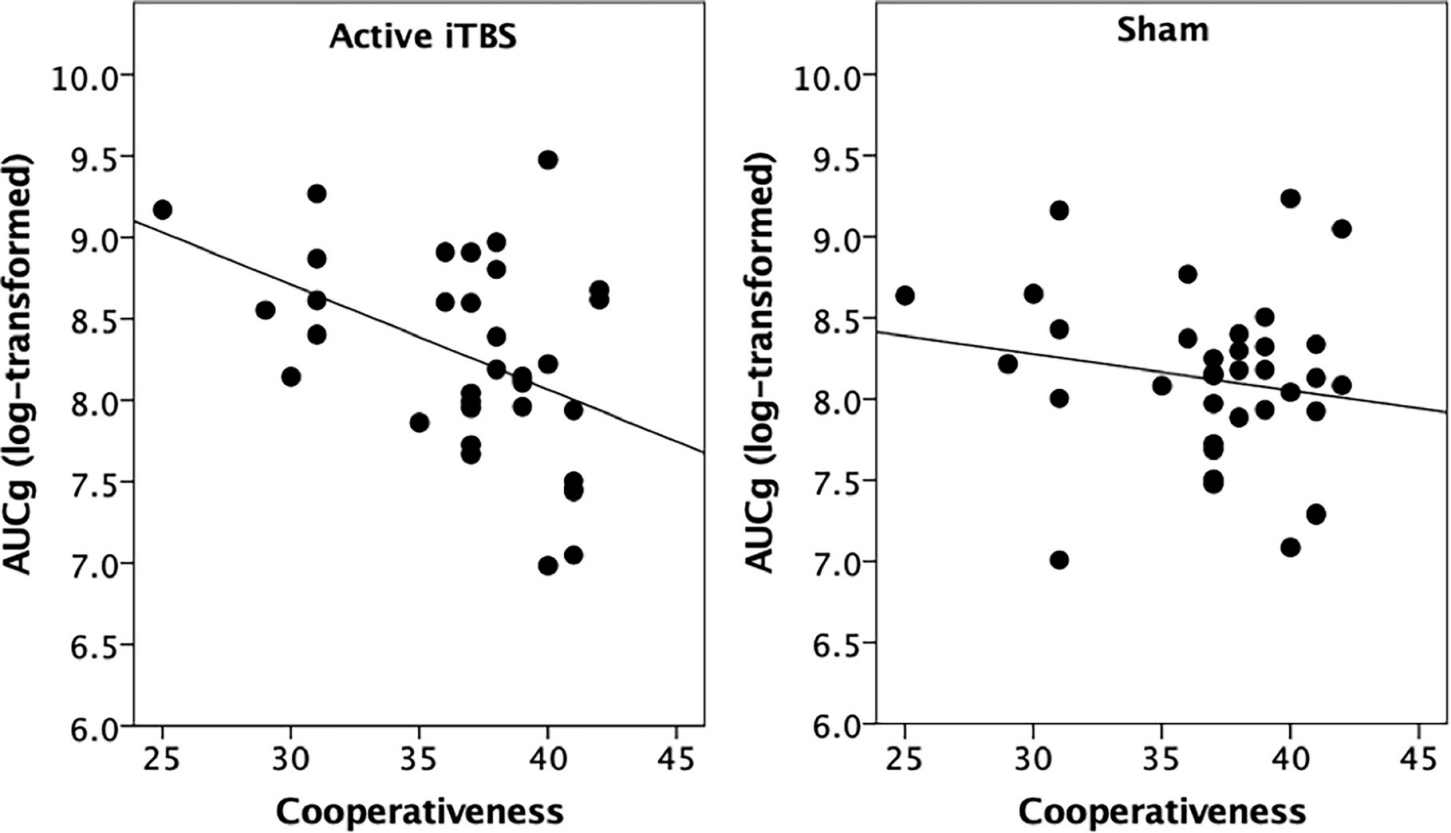
Scatterplots for the unadjusted relationship between cooperativeness and *AUCg* during the active-iTBS and sham-iTBS sessions. *AUCg* = Area under the curve with respect to the ground; iTBS = intermittent Theta Burst Stimulation.

To further clarify the meaning of the significant interaction effects with Cooperativeness and Self-Transcendence, we performed partial correlation analyses (controlling for the time when the participants started the session) to investigate the relationship between *AUCg* during the two iTBS sessions and the scores in both character dimensions. These analyses revealed that lower *AUCg* during the active iTBS was significantly related to higher Cooperativeness (*r* = -0.38, *p* = 0.03), but not during the sham iTBS session (*r* = -0.07, *p* = 0.72) (Fig 2). For self-transcendence, the correlations between this dimension and *AUCg* during the active and sham iTBS did not reach our statistical threshold (Active-iTBS: *r* = -0.32, *p* = 0.07; Sham-iTBS: *r* = 0.05, *p* = 0.77). These results indicate that women with higher scores in Cooperativeness display lower cortisol secretion during the active-iTBS sessions, but not during sham-iTBS.

Given that the participants reported being overall angrier and tenser during the first session when they received active iTBS, we repeated our analyses controlling for differences between stimulation sessions (active vs. sham iTBS) in anger and tension. Moreover, mood changes may have driven cortisol changes in some subjects. Therefore, we also repeated our analyses controlling for differences between stimulation sessions in changes in mood. As an index of changes in mood, we calculated the *AUCi* using a composite of the six VASs scales. The statistical conclusions of our study are the same if the analyses are performed controlling for differences in mood.

## Discussion

In this study, we investigated the influence of the TCI character dimensions on the effect of iTBS applied after a stressful situation on the cortisol secretion. Although we found overall no differences in total cortisol secretion (i.e., *AUCg*) or cortisol response to stress (i.e., *AUCi*) and stress-induced changes in mood between the active and sham iTBS sessions, we observed that higher scores in Cooperativeness were associated with lower cortisol secretion (i.e., *AUCg*) during the active, but not the sham iTBS sessions. None of the other temperament and character dimensions showed a significant effect.

First of all, we did not observe differences in HPA axis activity between the active and sham iTBS sessions. Although these results are not in line with our hypothesis, they agree with our former findings that in healthy females the application of rTMS to the left DLPFC does not significantly alter HPA-system functioning, by means of cortisol changes [26,36]. Furthermore, our results also indicate that in healthy volunteers not only one but also two successive iTBS sessions do not affect mood, as no significant differences in stress-induced changes in mood between the active and sham stimulation were observed (for a review see [62]). Moreover, the statistical conclusions are the same if the analyses are controlled for differences between sessions in mood (results not shown). Therefore, possible mood influences on the physiological response to stress could be excluded. These observations suggest that—at least in the healthy state—rTMS interventions do not affect the stress-response following a stressful event without taking into account interindividual differences. Indeed, our current findings indicate that certain personality dimensions may moderate the effect of TMS on the cortisol secretion after a stressful situation. When introducing individual personality information into the cortisol analyses, we found that the higher the scores on the character dimension Cooperativeness, the lower the cortisol secretion (i.e., *AUCg*) during the active, but not the sham iTBS sessions. Importantly, given that the time when the participants started the session was controlled for in the analyses, our results cannot be attributed to the circadian variation in cortisol. These observations support our former statements that individual differences modulate the stress response after the application of rTMS in healthy female subjects [28]. Importantly, in our previous study, we observed an effect of HF-rTMS before the stress task on *AUCi* [27]. In the current study, however, the effect of iTBS after the stress task was specific for *AUCg*. These observations indicate that rTMS over the left DLPFC may reduce the sensitivity of the HPA axis to stressful events when applied before the stressor, reflected in lower *AUCi*, and the intensity of the stress response in individuals scoring higher in Cooperativeness when rTMS is applied to a stressed brain, reflected in lower *AUCg* [50,51].

This study provides additional important evidence to understanding the inter-individual differences in the clinical effects of rTMS in major depression. Previous rTMS treatment studies have shown that Agreeableness and Extraversion, two dimensions of the NEO-V model of personality [11], predicted the clinical outcome of HF-rTMS and deep HF-rTMS on MDD patients [12,13]. Albeit the NEO-V and TCI may not measure the same construct, it is important to note that the Agreeableness and Extraversion dimensions of the NEO-V overlap with Cooperativeness [14]. Furthermore, Cooperativeness and Self-Directedness are often correlated (e.g., [14]); in the current study we observed a significant *r* = 0.4 association) and low scores in these two dimensions are considered a basic characteristic of major depression [63–65]. These results may explain why higher scores in Self-Directedness in treatment resistant depressed patients predicts clinical outcome after left DLPFC HF-rTMS treatment in our previous study [10]. Importantly, it has been proposed that the effect of rTMS in MDD may occur at the hypothalamic level, by inhibiting cortisol releasing hormone synthesis and release [21]. Along this line, results of our fundamental study in healthy volunteers suggest that the reason Cooperativeness and Self-Directedness predict the response of rTMS treatment in MDD [10] is because rTMS reduces cortisol secretion in individuals high on these dimensions. Further research is needed to understand the underlying neurobiological mechanisms explaining the influence of this character dimension to the effects of excitatory rTMS on cortisol secretion.

In a recent study, Siddiqi et al. [15] observed that persistence predicted antidepressant response to rTMS treatment. In the current study including only healthy female volunteers, however, persistence did not influence the effects of iTBS on the activity of the HPA axis. One possible explanation for these results could be that the influence of persistence on rTMS treatment observed in Siddiqi et al. [15] would not be driven by the effect of rTMS on the HPA axis, and as proposed by the authors persistence may affect rTMS outcomes due to its relationship with baseline left-hemispheric cortical reactivity. However, it is important to note that we investigated the effect of rTMS on the HPA axis in healthy female participants only, whereas Siddiqi et al. [15] focused on treatment response in depressed patients. More research is needed to investigate whether TCI subscales may also be related to the effect of rTMS on HPA axis activity in a clinical population.

Despite the novel findings, some limitations should be considered. In this study, only healthy young women using hormonal contraceptives were included. The use of a homogeneous sample allows us controlling for the possible effect of age, sex and the menstrual cycle on the activity of the HPA axis and cortisol secretion under stressful situations [33], and on the effect of rTMS [66]. However, this may reduce the generalizability of our results, and more research is needed in different populations. Besides that the order of the stress tasks differed (before or after stimulation), and that two instead of one stimulation sessions were applied, HF-rTMS and iTBS may result in different neurophysiological effects [67], and therefore, our results could not be directly comparable with previous studies using HF-rTMS. Another limitation of the study is the number of statistical analyses performed. Although we had specific hypotheses regarding the influence of temperaments and characters, we cannot exclude the possibility of type I error in our findings. Therefore, more research is needed to replicate the results of this study. Finally, although we used a sham coil that mimics the auditory and physical sensations of the active stimulation, we did not measure scalp pain and physical discomfort during the stimulation, and we cannot directly control for their effect on our results. However, it is important to note that, if the active iTBS provoked more pain or discomfort the participants, we could expect influences on negative affect. Our results showed that changes in mood were similar during both sessions and, most importantly, the statistical conclusions remain the same if we perform the analyses controlling for mood (results not shown). Furthermore, when asked at the end of the experiment, most of the participants (73.5%) could not discriminate between the sham and active iTBS sessions. Together, these results indicate that our findings would not be due to differences between the two sessions and to possible learning effects due to the use of a within-subject design.

In conclusion, our results show that when two sessions of iTBS over the left DLPFC are applied in stressed healthy females, a reduction in cortisol secretion is observed in individuals scoring higher in Cooperativeness, a character dimension of the TCI inventory. Our observations provide relevant evidence to the idea that inter-individual differences in personality factors may have an influence on the effects of rTMS. Finally, our findings shed further light on the understanding of the influence of personality characteristics in the clinical outcome of rTMS in stress-related disorders.
